# The pacific chapter annual meeting of the undersea & hyperbaric medical society

**DOI:** 10.1186/2045-9912-1-19

**Published:** 2011-08-04

**Authors:** Robert P Ostrowski, Takkin Lo, John H Zhang

**Affiliations:** 1 Department of Physiology and Pharmacology, Loma Linda University School of Medicine, Loma Linda, CA, USA; 2 Department of Internal Medicine, Loma Linda University School of Medicine, Loma Linda, CA, USA; 3 Department of Neurosurgery, Loma Linda University School of Medicine, Loma Linda, CA, USA; 4 Department of Anesthesiology, Loma Linda University School of Medicine, Loma Linda, CA, USA

## Abstract

The following is the summary report on the UHMS Pacific Chapter Annual Meeting held in Long Beach in October 2010. The conference provided the latest updates on scientific, technical and organizational aspects of Hyperbaric and Diving Medicine. Invited speakers gave series of lectures dealing with current standards of clinical practice and presenting the results of laboratory investigations with particular emphasis on mechanisms of hyperbaric oxygen therapy. Scientific sessions were accompanied by vendor exhibits and social events.

## Introduction

Many hyperbaric medicine practitioners, research scientists and health care providers attended the Pacific Chapter Annual Meeting of the Undersea and Hyperbaric Medical Society held in Long Beach California between October 15 and 17 (Friday through Sunday) in 2010. In the splendid interior of Hyatt Regency took place a very informative conference updating participants on the latest developments yet building on well established concepts in the field.

The 2010 annual meeting was chaired by the President of UHMS Pacific Chapter Dr. Stuart Miller, hyperbaric medicine specialist from Long Beach Memorial, and an expert in wound care.

There were several accompanying events and attractions including exhibits, a lottery with tickets to the Aquarium of the Pacific and hyperbaric medicine books as prizes, and UHMS Pacific Chapter Executive Committee Meeting.

It was a good meeting that helped to span the gap between basic sciences and hyperbaric medicine practice. The meeting offered also in depth insight on new applications of hyperbaric oxygen and the mechanisms of treatment. Those three memorable days at Long Beach were filled with exchange of opinions, vigorous discussions and social activities. The presented material could be classed into the following categories: 1) expository lectures on hyperbaric medicine applications in diving and in different medical conditions including wound care, osteomyelitis, plastic surgery problems, cardiovascular diseases, and organ transplant, all intertwined with basic science data and the overview of experimental and clinical studies; 2) case presentations with literature review; 3) health insurance issues with regard to HBO treatment, the logistics of hyperbaric medicine procedures and specialty information.

## Hyperbaric Medicine Lectures

On Friday, hyperbaric medicine lectures began with an interesting presentation by Dr. Karen Van Hosen from UCSD. She gave an overview of multiple factors that determine physiological phenomena and treatment outcomes in decompression sickness. Those included nitric oxide, heat shock proteins, cold stress and genetic susceptibility.

On the very same day came out an interesting presentation on medications in diving provided by Dr. Nicholas Bird, chief medical officer of Divers Alert Network. Dr. Bird laid out physician's views on evaluation of divers who take medications. The safety of diving on meds remains an issue somewhat unresolved. Many medications have been found unconnected to diving risks, however medications as markers of chronic conditions surely indicate that diver's fitness to dive may be compromised [1]. Special concerns have been voiced over sedatives and anxiolytics as well as narcotic pain medications. These meds can impair the performance of the diver by impairing physical fitness/exercise capacity or mental status.

On Saturday Dr. Garret Wirth gave a talk on hyperbaric oxygen therapy in plastic and reconstructive surgery. Dr. Wirth is a wound care specialist at Long Beach Memorial and a plastic surgeon at UCI. After presentation of graphic images of bodily defects and mutilations seen by reconstructive surgeons in their practice, Dr. Wirth evoked a description of Dantes' 8th circle of hell and physical deformities sustained by its inhabitants. The presentation was further unfolded to offer an overview of plastic/reconstructive surgery techniques and HBO applications for improving survival of flaps and grafts [2]. As it was stressed normal flaps or grafts with uncompromised blood supply do not require HBO however this modality should be used when there is a documented perfusion concern or vascular insult [3]. In addition this treatment is usually done after surgical measures have been considered. The animal studies and clinical reports have also shown that HBO increases the length of vessel growth in pedicled flaps [3,4]. Dr. Wirth offered examples of how he uses HBO in his practice of plastic and reconstructive surgery. Flap deterioration, compromised replants, wounds in the irradiated fields, wounds with polymicrobial infections and after crush injury comprised the majority of presented indications. He also pointed out that the use of HBO for cosmetic surgery has been recently compromised by bad economy.

The following was hyperbaric literature update presented by Dr. Dick Clarke. He began his informative talk with discussing a report on facilitated chronic foot ulcers healing in the diabetic patients with HBO therapy [5]. Dr. Clarke outlined the criteria of enrollment and randomization and presented the outcomes. This noteworthy study, published in Diabetes Care, adopted a double blinded design and followed the rules of randomization. Londahl and collaborators were able to evidence significantly improved indexes of foot healing in the HBO group over placebo. Study design, as pointed out by Dr. Clarke, adheres to CONSORT criteria [6]. Dr. Clarke was able to identify only a few insufficiencies of the study including the lack of transcutaneous oximetry monitoring in the course of therapy [7]. However, he also presented the counterpoint to findings favorable for HBO, published as an editorial by Dr. Berendt in Clinical Infectious Diseases [8]. According to Dr. Berendt's view the evidence is not strong enough to conclude that HBO is effective in the treatment of chronic foot ulcers in diabetic patients. One of the major goals of HBO therapy is to avoid amputation in such patients. The study by Londahl did not show clinical benefit with this regard however other authors reported that amputation rate decreased in HBO treated patients [9,10]. Dr. Clarke postulated that conducting large controlled randomized double blinded trials should be done before consensus can be reached on this issue. Dr. Clarke further stressed the need to monitor tissue oxygen concentration in patient undergoing HBOT. The rise in tissue oxygen pressure may be insufficient in patients undergoing HBO therapy and this inadequate response can lead to healing failure. Moreover low pt O_2 _values in the course of therapy allow to predict which wounds will not heal [11,12]. Next, Dr. Clarke discussed the latest literatures on the use of HBO in the necrotizing soft tissue infections (NSTI). A recent study has demonstrated that HBO did not show clinical benefits in NSTI despite aggressive treatment regimen (consistent with the treatment of gas gangrene) [13]. In another discussed paper, however, Hassan and coworkers from Burn Center at Augusta found a strong tendency towards reducing amputation in patients with necrotizing fasciitis treated with HBO [14]. Dr. Clarke also presented the results of clinical investigations that revealed improved quality of life in patients treated with HBO after radiation of head and neck tumors. The treatment regimen comprised 30 sessions of hyperbaric oxygen at 2.5 ATA. The total radiation dose received by patients in that study was 46 Gy over 15 days, which is significant [15]. Dr. Clarke pointed out that the study was underpowered and lacked blinded protocol. He also raised ethical concern over quality of life in the untreated controls as an outcome and over patients' consent to suffering. Other studies dealing with HBOT for osteoradionecrosis (ORN) seem to provide conflicting results. For example the data published by Dr. Annane's group in 2009 do not seem to build a strong case for HBO use in ORN [16,17]. Dr. Clarke discussed several factors that could weigh in on the quality of this study and authors' conclusions. Although most studies suggest beneficial role of HBO in ORN, further research is needed to validate use of HBO against radiation induced injuries [18-20]. Then the audience was updated on HBO administration in cardiovascular diseases. Dr. Yogaratnam and colleagues preconditioned patients with HBO for two 30-minute intervals at 5 minutes apart on the morning of coronary artery bypass grafting (CABG) [21]. HBO preconditioning (HBO PC) improved left ventricular stroke work in these patients and decreased their length of stay in ICU. In addition, smaller rise in serum troponin level was noted in HBO PC group. The authors advocated larger trial with improved scientific rigor. Dr. Clarke praised promising results of this study and recalled earlier reports from this group successfully using HBO PC for patients awaiting CABG with cardio pulmonary bypass [22].

Next presentation, done by Dr. Robert Ostrowski from Loma Linda University detailed the effect of hyperbaric oxygen treatment on neural tissue. He reviewed basic research that has been done in the past couple of years. The large portion of results originated in the lab of Dr. John Zhang who is a world renowned expert in experimental hyperbaric medicine. Dr. Ostrowski emphasized superiority of hyperbaric oxygen over normobaric oxygen therapy demonstrated in the rodent models of stroke. Part of the presentation was dealing with recent discoveries of molecular pathways that underlie HBOT action. New treatment strategies that combine HBO with pharmacological agents (including JNK inhibitors) to increase neuroprotective effect were one of many issues attracting audience attention [23]. Experimental studies on surgical brain injury and global cerebral ischemia ascribed increasing role in mediating HBO mechanisms preconditioning (HBO-PC) to cyclooxygenase 2 (COX-2) [24,25]. Another candidate mechanism of HBOT preconditioning postulates the exhaustion of the extracellular proteolysis [26]. Dr. Ostrowski also cited recent finding revealing that HBO can induce expression of nuclear factor E2-related factor-2 (Nrf2), which is a key regulator in reducing oxidative stress, inflammation and accumulation of toxic metabolites in cerebral tissues [27]. However, further studies are needed to address to what extent newly implicated pathways are involved in HBO mechanisms. Undergoing studies also point towards an important role of HBO in the activation of neurogenesis, which carries significant clinical potential for regeneration of the brain after major stroke associated with tissue loss [28-30]. During discussion that followed some participants raised concerns over short regimens of experimental HBO treatment that might not be intensive enough to provide significant improvement in the clinical setting. The discussion also touched upon theoretical foundations of HBO combined with pharmacological agents or with other preconditioning modalities such as hypothermia [31] followed by questions of optimal timing for combined treatment in the clinical setting.

During another interesting presentation Dr. Ostrowski laid out historical background and summarized recent advancements in the use of HBO for organ transplant. HBO have been used in a variety of clinical and experimental transplantation procedures involving intestine, pancreas and liver [32-35]. HBOT was found to improve graft survivability and function in the experimental setting [36]. Laboratory investigations revealed several mechanisms of how HBO benefits transplanted organs including disruption of hypoxic signaling reflected by a decreased level of hypoxia inducible factor 1 α, as found in transplanted syngeneic pancreatic islets [37]. Dr. Ostrowski reviewed progress in the use of HBOT for preservation of organ transplants including HBO pretreatment of donors [38]. In the discussion that ensued hyperbaric medicine practitioners pointed out that HBO researchers need to further address usefulness of HBO treatment for allogenic transplants. Dr. Stuart Miller suggested testing the impact of HBO on the transplant triggered immunologic response and HBO interactions with immunosuppressants commonly administered to transplant recipients.

Then Dr. Michael Strauss from Long Beach Memorial took the floor with arch interesting lecture dealing with refractory osteomyelitis. He presented definition and pathophysiology of osteomyelitis, the inflammation of the bone and bone marrow, with clinical presentations and HBO treatment protocols. More than ever HBO is used for refractory osteomyelitis [39]. As one of the major mechanisms of HBOT in osteomyelitis Dr. Strauss indicated increased intramedullary oxygen tension to the point where killing microbial organisms by WBC becomes effective. Earlier studies have shown that HBO in osteomyelitis was as effective as cephalexin however there was no additional benefit from HBO/antibiotic combo [40]. Moreover, HBO is bactericidal, and has been also shown to stimulate osteoclasis and angiogenesis [41]. More importantly HBO therapy produces good outcomes in over 80 percent of patients with osteomyelitis [42]. Dr. Strauss also evaluated clinical tests for diagnosing osteoradiomyelitis and suggested cautious interpretation of MRI which can give 50% false positives. Finally, he addressed academic integrity issues, which might affect the quality of results in some studies in this field.

Dr. Strauss continued lecturing on Sunday by outlining "reasons problem wounds fail to heal". According to Dr. Strauss, diabetic foot wound healing is compromised by a triad of factors formed by unresolved infection, hypoxia, and deformities. Dr Strauss addressed clinical management of all these conditions including use of HBO with aid of illustrations and clinical documentation. HBO ameliorates healing through several factors, including reduced edema in the wound, increased blood oxygen content and improved oxygen diffusion [43,44]. HBO-induced blood hyperoxygenation more than compensates reduced flow from vasoconstriction [45]. Dr. Strauss also pointed out that HBO drastically reduced amputation rates in a presented group of patients.

## Case Presentations

Series of interesting case presentations were given by Drs. Jeff Sipsy, Tom Ascuito, and Enoch Huang on Friday and on the following day by Drs. Susan Sprau, Ralph Potkin and Roxanna Sadri.

Friday case presentations included Dr. Enoh Huang's talk revealing the history of 68 old woman with TRAM flap applied after lateral mastectomy. The patient condition was deteriorating as the flap, showing skin color changes, was dying. The patient received a total of 20 dives at 2 ATA of 90 minute duration (BID). The photographic documentation confirmed that the flap showed remarkable amelioration under HBO treatment. Dr. Huang discussed mechanisms of HBO therapy for flap survival including the inhibition of leukocyte endothelial intravascular adhesion and direct oxygenation of ischemic tissues by increasing oxygen dissolved in plasma. In this mechanism HBO is effective even when the microcirculation is occluded to the point that RBC cannot pass through capillaries [46].

On the following day Dr. Susan Sprau from UCLA shared her experience from treating the acute postsurgical optic neuropathy. The patient, 29 yr old male, presented with cystic mass in the left orbit that was later diagnosed as schwannoma. The tumor was surgically removed. Patient's vision deteriorated after surgery from normal acuity to merely light perception, however. Optic nerve edema was present on physical exam. The patient was treated with steroids and HBOT modified navy TT5 then TT9 [47]. During the initial several days of treatment, vision improved at depth and deteriorated at the surface. Total number of HBOTs was 19, administered over 12 days. This treatment resulted in amelioration of swelling and improved visual acuity 20/25 with normal color testing. Dr. Sprau concluded that vision loss after ophthalmologic surgery may favorably respond to HBOT [48].

In turn Drs. Roxana Sadri and Emi Latham from UCSD hyperbaric medicine center presented a case of iatrogenic embolism. 58 yr old woman with myofascial pain syndrome, paresthesia and the paralysis in the right arm and leg after radiofrequency thermoablation was initially treated with USN TT6 followed by Table nine on day 3,4 and 5 [49]. The authors noted significant sensory and motor improvement in the patient although the residual deficit forced her to walk with a cane. Dr. Sadri also discussed two cases of intramedullary gas bubbles secondary to epidural injections. She summarized routes of gas invasion and clinical treatment regimens [50].

## Logistic, Occupational, and Health Insurance Aspects in Hyperbaric and Diving Medicine

Several UHMS meeting presentations were dealing with logistic of running HBO facilities, health insurance requirements for reimbursing HBO therapy as well the education of hyperbaric medicine practitioners. On the first day of the conference Dr. Enoh Huang was talking on hyperbaric medicine subspecialty and gave us an update on hyperbaric medicine fellowships. Also on that day Dr. Nicolas Bird provided a presentation on Divers Alert Network (DAN). It is a very useful enterprise, with frequently updated website providing industrial and individual divers with tangible information. DAN promotes research activity to investigate questions regarding health and safety of divers. Quite recently Dr. Nicholas Bird filled its full-time chief medical officer position to oversee network's mission. One of the ongoing projects there investigates potential seizure threshold lowering effect of sudafed in the HBO environment [51]. This study, being conducted in collaboration with Dr. Jay Dean as a PI was launched in 2010 at the University of Florida. The aim of another ongoing study is to compare diving performance of the individuals with patent foramen ovale (PFO) versus those with medically closed foramen ovale. The foramen ovale situated in the interatrial septum allows blood to circumvent pulmonary circulation in utero [52]. The closure of foramen ovale is not complete in 25% adults hence its significant prevalence amongst divers. PFO is not a threatening condition in the general population however in divers it can pose a risk of an arterial gas embolism in decompression illness (gas bubbles pass directly into the left atrium bypassing the lungs). DAN investigators are currently working to establish the criteria for closing foramen ovale that would take into account past history of decompression sickness (DCS), presence of IE or neurological DCS, size of foramen ovale and required level of diver's performance. DAN website offers an interesting selection of diving medicine articles: http://www.diversalertnetwork.org. Dr. Bird also discussed needs of veteran members of DAN. With the help from epidemiology PhD students, the complete survey of divers is underway. It is "aimed at establishing the prevalence of cardiovascular risk factors, diabetes, asthma, access to health care and diving practice". Interestingly, divers appear to have less chronic symptomatic diseases than in the general population. DAN investigators also pointed out that age itself is not a causative factor for death in known diving accidents and identified major triggers of death as insufficient gas, entrapment and equipment problems [53].

Also on the first day of the meeting Dr. Karen Van Hosen gave a talk on how to establish diving medicine clinic and evaluate recreational, scientific and commercial divers for clearance to dive.

Another Friday talk provided an overview of diving computer applications. It was presented by Dr. Karl Huggins from Catalina hyperbaric chamber at the University of Southern California. Dr. Huggins covered the basics then moved on to specify what data are collected by diving computes (depths, times, warnings, decompression status, temperatures and dive profiles) and delineated data acquisition modes [54]. This was followed by examples of dive computer recordings and their interpretation, including analysis of data recovered from fatalities.

The hyperbaric medicine practice and coding formed the title of presentation provided by Dr. Dick Clarke on Saturday. In the course of his talk Dr. Clarke laid out the strategy of how to handle HBO treatments for reimbursement purposes. One of ways of dealing with this issue is to seek pre-authorization to treat. Unfortunately some insurers specifically indicate that use of HBO is not reimbursable for prophylactics of certain conditions such as mandible osteoradionecrosis. Dr. Clarke provided the points that can bolster argumentation that use of HBO is medically necessary [55]. Interesting presentation was also delivered by Dr. Ralph Potkin who shared his experience from work at the free-standing HBO facility. Dr. Potkin presented the statistics of treated cases and offered a logistic insight on managing the facility.

With great interest the audience welcomed a Sunday presentation given by Dr. Kim Buschmann from the Canadian Navy who is also an instructor of hyperbaric medicine at UCSB. She presented tasks, risks and problems experienced by technical divers including nitrogen narcosis. Dr. Buschmann offered an overview of decompression schedules. The presentation gave everyone quite an insight into respiratory physiology of diving addressing issues such as work of breathing and impact of increased density of gas on respiratory dead space, and the effects of hypercapnia, hyperoxia and hypoxia on respiratory physiology parameters [56]. Dr. Buschmann also addressed problems of inner ear decompression including selection of decompression gasses for treatment [57,58].

An interesting lecture on hyperbaric oxygen chamber safety in a large urban healthcare facility was given by Dr. David Stiles. Dr. Stiles briefly presented the history of Long Beach Memorial opened in 1907 as Seaside Hospital. Its Hyperbaric Medicine Department has 6 monoplace treatment chambers and in its 30 year history treated 20,000 plus patients. Dr. Stiles provided an overview of procedures at hyperbaric facility, identified prospective areas of innovation, and addressed problems that may arise during standard operating procedures. This presentation was followed by Dr. John Kades' talk, focusing on the guidelines of forensic investigations of diver deaths.

## Future Studies and Conclusions

Presentation of many novelties and often highly experimental approaches frequently led to controversy that stirred vivid discussion on the future directions for hyperbaric medicine. Certain case reports raised criticism of other hyperbaric medicine practitioners over too conservative treatment. During the meeting, several participants were trying to work out the consensus between basic science results and clinical practice in hyperbaric medicine. They stressed the existing gap between these two fields and postulated to increase translatability of laboratory investigations. In particular Dr. Miller addressed the paucity of results on several important molecular and cellular aspects of HBOT in organ transplant studies and in wound care research. Participants discussed the use of HBOT in combination with pharmacological agents and personalized treatment regimens. The need to develop new molecular markers for monitoring effects of HBO therapy also emanated from the discussion. Many participants postulated to increase scientific rigor of experimental and clinical studies with HBOT for improved quality of results and academic excellence.
